# Supplementary material for: Evidence for pressure induced unconventional quantum criticality in the coupled spin ladder antiferromagnet C9H18N2CuBr4
Source: Nat Commun. 2022 Jun 2;13:3073. doi: 10.1038/s41467-022-30769-8 (PMC9163114; doi:10.1038/s41467-022-30769-8)
Supplement: Supplementary file 1 — Supplementary Information [file 41467_2022_30769_MOESM1_ESM.pdf]

Supplementary Information for Evidence for pressure induced unconventional quantum criticality in the coupled spin ladder antiferromagnet $C_9H_{18}N_2CuBr_4$

Tao Hong,[1] Tao Ying,[2] Qing Huang,[3] Sachith E. Dissanayake,[4] Yiming Qiu,[5] Mark M. Turnbull,[6] Andrey A. Podlesnyak,[1] Yan Wu,[1] Huibo Cao,[1] Yaohua Liu,[1,7] Izuru Umehara,[8] Jun Gouchi,[9] Yoshiya Uwatoko,[9] Masaaki Matsuda,[1] David A. Tennant,[3,10] Gia-Wei Chern,[11] Kai P. Schmidt,[12] and Stefan Wessel[13]

[1]*Neutron Scattering Division, Oak Ridge National Laboratory, Oak Ridge, Tennessee 37831, USA*
[2]*School of Physics, Harbin Institute of Technology, 150001 Harbin, China*
[3]*Department of Physics and Astronomy, University of Tennessee, Knoxville, Tennessee 37996, USA*
[4]*Department of Physics, Duke University, Durham, North Carolina 27708, USA*
[5]*National Institute of Standards and Technology, Gaithersburg, Maryland 20899, USA*
[6]*Carlson School of Chemistry and Biochemistry, Clark University, Worcester, Massachusetts 01610, USA*
[7]*Second Target Station, Oak Ridge National Laboratory, Oak Ridge, Tennessee 37831, USA*
[8]*Department of Physics, Yokohama National University, Yokohama 240-8501, Japan*
[9]*Institute for Solid State Physics, University of Tokyo, 5-1-5 Kashiwanoha, Kashiwa, Chiba 277-8581, Japan*
[10]*Department of Materials Science and Engineering, University of Tennessee, Knoxville, Tennessee 37996, USA.*
[11]*Department of Physics, University of Virginia, Charlottesville, Virginia 22904, USA*
[12]*Lehrstuhl für Theoretische Physik I, Staudtstrasse 7, Universität Erlangen-Nürnberg, D-91058 Germany*
[13]*Theoretische Festkörperphysik, JARA-FIT and JARA-HPC, RWTH Aachen University, 52056 Aachen, Germany*

Contents

Supplementary Note 1: Reversibility of the phase transition in DLCB under pressure 1

Supplementary Note 2: Analysis of the scaling behavior under pressure in DLCB 2

Supplementary Note 3: Analysis of broadening effect attributed to spontaneous magnon decays 2

Supplementary Note 4: Crystal structure and magnetic interactions under pressure 4

Supplementary Note 5: Single-crystal neutron diffraction measurements under pressure 5

Supplementary Note 6: Single-crystal inelastic neutron measurements under pressure 6

References 12

Supplementary Note 1: Reversibility of the phase transition in DLCB under pressure

It should be noted that in order to increase or decrease the force on the piston, i.e., change the hydrostatic pressure, the clamp-type pressure cell has to be removed from its experimental setup. The following procedure was set up to test reversibility of the phase transition in DLCB under pressure: Firstly, the AC heat capacity at ambient pressure on a deuterated single-crystal sample was measured by the Physical Property Measurement System (PPMS). After that, the sample was loaded to the pressure cell and the AC heat capacity was performed at 0.38 and 1.0 GPa, respectively, in a dilution refrigerator. The next pressure was targeted for 0.38 GPa but the actual pressure was 0.51 GPa due to the fact it is difficult to control the desired pressure while releasing the pressure. After the measurement at 0.51 GPa, the pressure was completely relieved and the sample was taken out from the pressure cell. Finally, the measurement at ambient pressure was repeated by PPMS. The overall results are summarized in Supplementary Fig. 1. At ambient pressure and 0.38 GPa, an anomaly, which indicates a phase transition to the Néel ordered state, is clearly visible. It disappears at 1.0 GPa but was restored at 0.51 GPa and ambient pressure in the decompression run. The observed

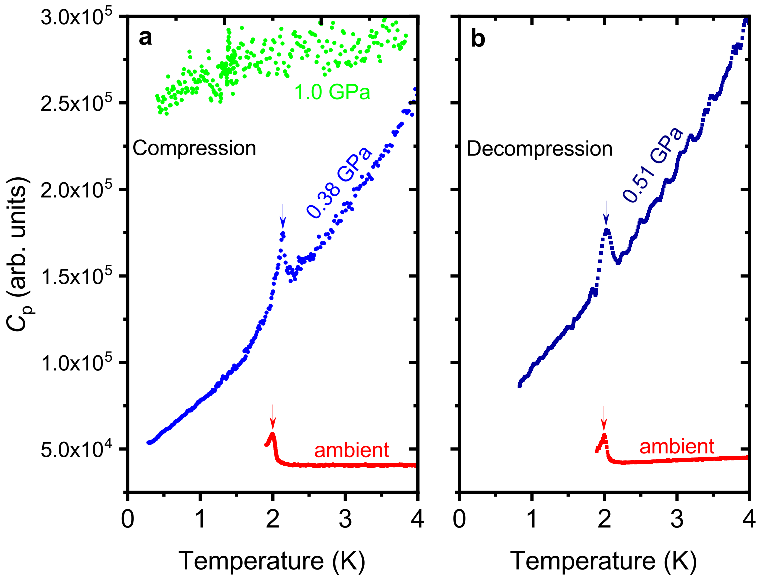

Supplementary Figure 1: **The AC heat capacity $C_{\rm P}$ of DLCB as a function of temperature.** **a** At ambient pressure, 0.38, 1.0 GPa in the compression run. **b** At 0.51 GPa and ambient pressure in the decompression run. For clarity, the data are shifted upwards. The transition temperature is indicated by an arrow.

transition temperatures with pressure are consistent with the phase diagram as shown in Fig. 1 of the main article. Thus, we can conclude that the pressure-induced phase transition in DLCB is reversible.

Supplementary Note 2: Analysis of the scaling behavior under pressure in DLCB

Figure 2(d) in the main article shows the determined pressure dependence of the ordered moment size m and the best fit to a power law $m \propto (P_{\rm c} - P)^{\beta}$ yields $P_{\rm c}$=1.04(4) GPa and the order-parameter exponent β=0.68(5). Here, we perform a careful examination to estimate the quantum-critical regime in which the general power law can be extracted. Following the similar procedure as described in Refs. [1, 2], we evaluate the critical exponent β from fitting the power law to the different size of the pressure window $p_{\rm w}$ near the quantum critical point (QCP), where $p_{\rm w}$ is the maximum of the reduced pressure $(P_{\rm c}$-$P)/P_{\rm c}$ of the fit window. Supplementary Fig. 2 shows the variation in β with $p_{\rm w}$. The linear extrapolation gives β=0.675 as $p_{\rm w}\rightarrow 0$, which is identical to β at $p_{\rm w}$=1. Consequently, we can conclude that the scaling behavior $m \propto (P_{\rm c} - P)^{\beta}$ can be applied for the entire investigated pressure region in DLCB, and so can the scaling behavior $\Delta_{\rm LM} \propto (P_{\rm c} - P)^{\nu z}$ in the same critical regime. This is consistent with the fact that DLCB is already in the vicinity of the QCP at ambient pressure.

Supplementary Note 3: Analysis of broadening effect attributed to spontaneous magnon decays

Magnetic excitation spectra of DLCB in the quantum disordered (QD) phase show a gapped broad excitation continuum. Here, we discuss the possible cause due to the spontaneous decay of the one-magnon state into the multi-magnon continuum by quantum fluctuations of interacting magnons. For instance, the process of one-magnon decays into the two-magnon continuum is allowed if the following kinematic conditions are satisfied:

$$\mathbf{q} = \mathbf{q}_1 + \mathbf{q}_2, \tag{1}$$

$$\varepsilon_2(\mathbf{q}) = \varepsilon_1(\mathbf{q}_1) + \varepsilon_1(\mathbf{q}_2), \tag{2}$$

$$\varepsilon_2(\mathbf{q})^{\rm min} \leq \varepsilon_1(\mathbf{q}) \leq \varepsilon_2(\mathbf{q})^{\rm max}, \tag{3}$$

where ε_1 is the one-magnon dispersion relation, and $\varepsilon_2^{\rm min}$ and $\varepsilon_2^{\rm max}$ are the lower and upper bounds of the two-magnon continuum, respectively.

At the critical point, the longitudinal mode (LM) becomes gapless whereas the transverse mode (TM) remains spin-gapped as 0.44(3) meV due to an Ising-type exchange anisotropy. Supplementary Fig. 3(a) shows quantum

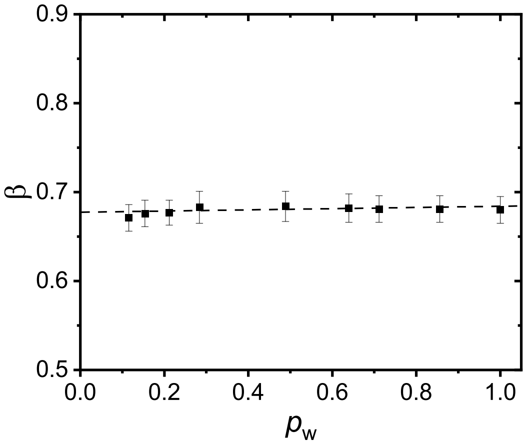

Supplementary Figure 2: **The order-parameter exponent β as a function of p_w.** Estimation of the critical exponent β from fitting the general power law to the different size of the pressure window p_w near the quantum critical point. The dashed line represents linear regression. Error bars represent one standard deviation.

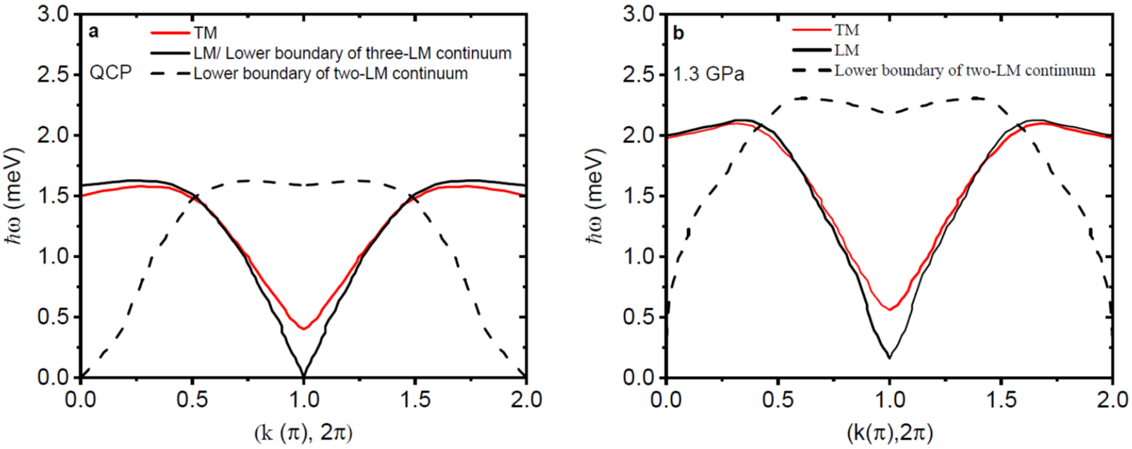

Supplementary Figure 3: **Quantum Monte Carlo simulation of magnetic excitations in the quantum disordered phase.** Quantum Monte Carlo results of the transverse mode (TM) and longitudinal mode (LM) along the ladder direction overplotted with the lower boundary of multi-particle continuum **a** at the quantum critical point (QCP) and **b** at P=1.3 GPa.

Monte Carlo (QMC) calculations of TM, LM and the lower boundary of two- and three-LM continua which were obtained from the dispersion relation of LM. At the antiferromagnetic wavevector $\mathbf{k}$=(π,2π), the lower boundary of two-LM continuum lies well above the energy gap of TM and therefore the spontaneous decay of TM into a pair of LMs is kinematically forbidden. Although decays into the three-LM continuum are kinematically allowed in DLCB, the effect is expected to be small for the following reason: the decay rate is related to the strength of anisotropic interactions [3] and the anisotropic DM interaction in DLCB is negligible as discussed in the main article. Beyond the critical point at 1.3 GPa, LM acquires a gap of $\Delta_{\rm LM}$=0.25(3) meV and $\Delta_{\rm TM}$ moves further up to 0.56(4) meV. In a similar situation to the critical point, the spontaneous decay of TM into a pair of LMs is kinematically forbidden at 1.3 GPa as shown in Supplementary Fig. 3(b). As three times of $\Delta_{\rm LM}$ is well above $\Delta_{\rm TM}$, the decay of the TM mode into the three-LM continuum is also forbidden due to the energy conservation.

Overall, the broadening effect due to multi-magnon decays can be rightly excluded. We want to emphasize that it holds true regardless of the assumption we made in the main article about no impact of applied pressure on the interaction anisotropy λ and the ratio between $J_{\rm rung}$ and $J_{\rm leg}$.

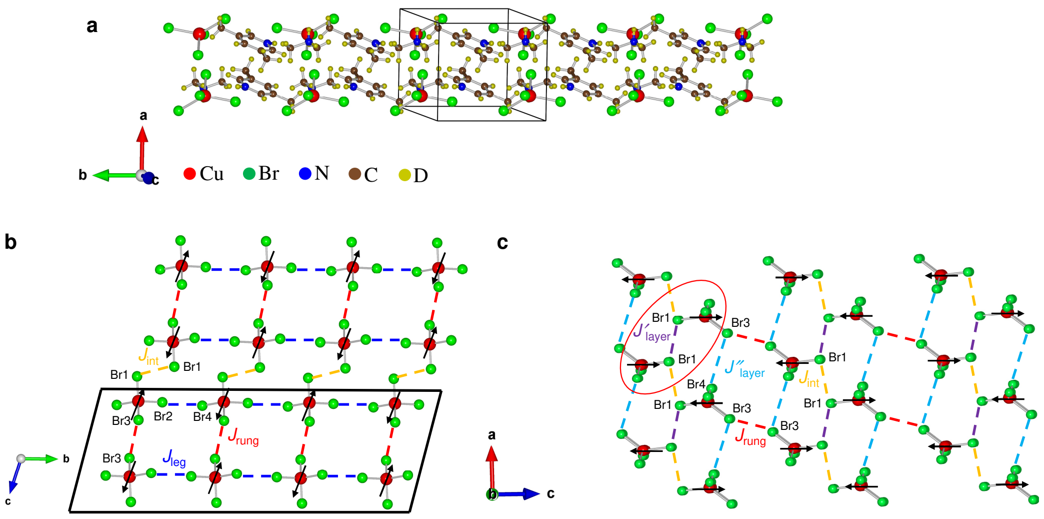

Supplementary Figure 4: **Crystal structure and magnetic interactions in DLCB. a** Crystal structure of deuterated $C_9H_{18}N_2CuBr_4$ projected along the crystallographic c-axis to show the stacking of discrete DMA^+ (C_2D_8N) and $35DMP^+$ ($C_7D_{10}N$) cations. Outlined is a nuclear unit cell. **b-c** Views of the five different exchange pathways considered in DLCB. The red, blue, yellow, purple and light blue dashed lines represent the superexchange pathways of $J_{\rm rung}$, $J_{\rm leg}$, $J_{\rm int}$, $J'_{\rm layer}$ and $J''_{\rm layer}$, respectively. The organic cations play no role in the magnetism and are not shown. The parallelogram outlines the two-leg ladder structure with the leg direction extending along the crystalline b-axis. Black arrows indicate the directions of the spins in the Néel ordered state. The ellipse outlines the frustrating inter-layer coupling $J'_{\rm layer}$ as discussed in the main article.

Supplementary Note 4: Crystal structure and magnetic interactions under pressure

The crystal structure of deuterated $C_9H_{18}N_2CuBr_4$ (DLCB for short) is triclinic in the space group $P\bar{1}$. Supplementary Fig. 4(a) shows the mixed-cation structures consisting of discrete DMA^+ and $35DMP^+$ cations, where DMA^+ is the dimethylammonium cation (C_2D_8N) and $35DMP^+$ is the 3,5-dimethylpyridinium cation ($C_7D_{10}N$). Nearest-neighbor and next-nearest-neighbor contacts between bromide ions suggest that ${CuBr_4}^{2-}$ anions form two-leg ladders along the crystallographic b axis. Additional single-crystal neutron diffraction data at several representative pressures were collected using a four-circle neutron diffractometer. The Rietveld refined unit cell parameters of DLCB at ambient and elevated pressures measured at T=5 K are summarized in Supplementary Tab. 1. It does not reveal any structural distortion at and above 1.06 GPa as the possible cause for the absence of magnetic order and the triclinic space group $P\bar{1}$ is preserved at each pressure. The Rietveld refined atomic coordinates, the isotropic displacement and occupancy factors are summarized in Supplementary Tabs. 2 to 5.

The networks of magnetic interactions due to S=1/2 Cu^{2+} ions in DLCB are mediated by the superexchange across the diamagnetic bromide ions *via* Cu-Br$\cdots$Br-Cu contact. The organic cations play no role in the magnetism. Supplementary Fig. 4(b) shows the schematic view of five superexchange pathways including the intra-ladder couplings $J_{\rm rung}$ and $J_{\rm leg}$, the inter-ladder coupling $J_{\rm int}$ and the couplings $J'_{\rm layer}$ and $J''_{\rm layer}$ between the two-dimensional layers in DLCB. Generally, the superexchange *via* the two-halide pathway depends upon the Cu$\cdots$Cu separation distance, Br$\cdots$Br contact distance and Cu-Br$\cdots$Br bridging angle. The shorter Cu$\cdots$Cu separation distance or Br$\cdots$Br contact distance and larger bridging angle lead to the stronger exchange interaction [4]. These values and their alteration at several representative pressures are summarized in Supplementary Tab. 6, which suggests that the application of pressure increases the strength of $J_{\rm leg}$ and $J_{\rm rung}$ whereas $J_{\rm int}$ decreases with pressure. Therefore, the applied pressure is effectively tuning the exchange coupling ratio $\alpha = J_{\rm int}/J_{\rm leg}$ thus driving the system from the Néel ordered state to a quantum disordered state. Supplementary Tab. 7 shows QMC calculations of the Hamiltonian parameters at different pressures.

With regard to the possible inter-layer coupling $J_{\rm layer}$, as listed in Supplementary Tab. 6, their Cu$\cdots$Cu separation distances become reduced under pressure whereas their Br$\cdots$Br contact distances initially grow with pressure and then decrease towards the critical point. They, therefore, can become pronounced in the vicinity of the critical point.

	ambient	0.88 GPa	1.06 GPa	1.3 GPa
a (Å)	7.454(11)	7.206(12)	7.178(13)	7.108(13)
b (Å)	8.268(12)	8.148(13)	8.083(14)	8.053(14)
c (Å)	13.755(12)	13.493(13)	13.435(13)	13.442(13)
α (°)	107.44(5)	107.18(5)	106.80(5)	107.12(4)
β (°)	90.43(5)	91.03(4)	91.22(4)	91.17(4)
γ (°)	91.00(5)	90.79(4)	90.33(5)	90.37(3)
R_F-factor	5.9	6.2	4.6	4.9
χ^2	3.9	5.6	3.0	4.0

Supplementary Table 1: Lattice parameters and unit cell volumes of deuterated $C_9H_{18}N_2CuBr_4$ listed for the space group $P\bar{1}$ measured at 5 K for several representative pressures.

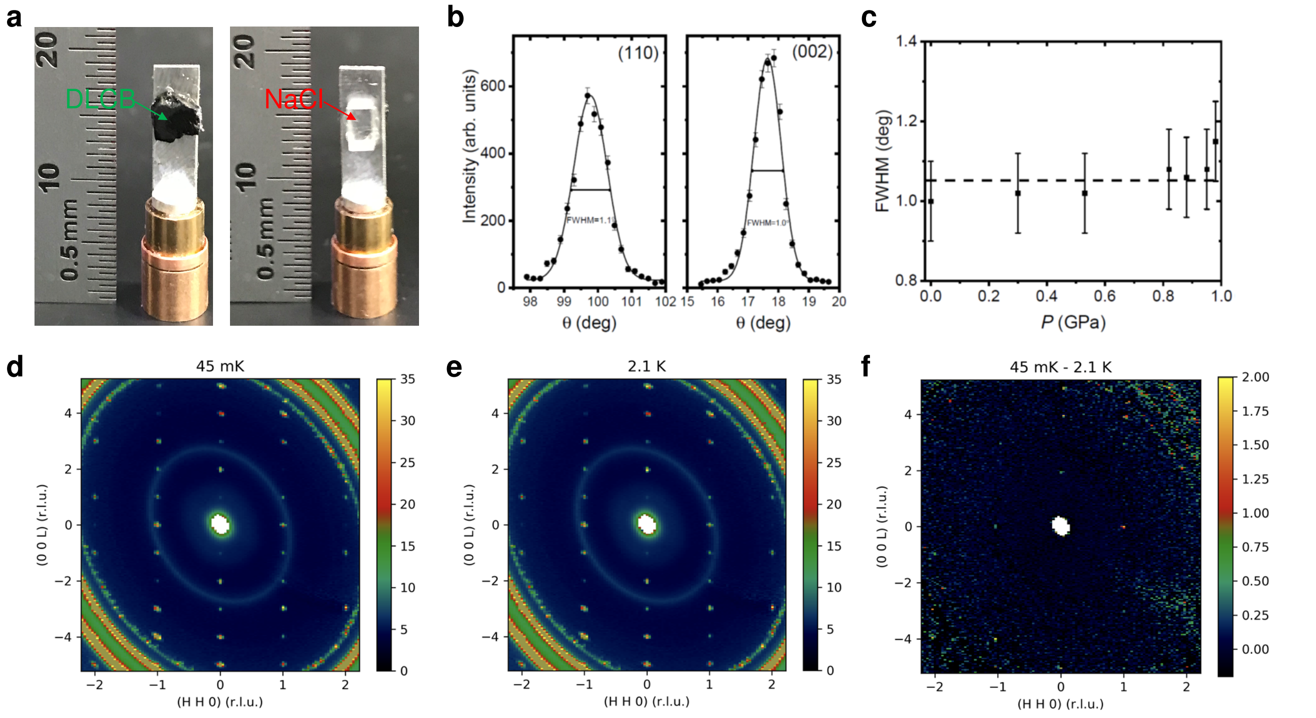

Supplementary Figure 5: **Single-crystal neutron diffraction measurements under pressure.** **a** Photographs of a deuterated single crystal of DLCB glued on the front side of an aluminum sample holder. A piece of NaCl single crystal was also mounted on the back side for calibration of pressure. **b** The rocking-curve scans of the nuclear Bragg reflections (1 1 0) and (0 0 2) measured at CTAX. Full width at half maximum (FWHM) of the overall sample mosaic is about 1°. **c** Pressure-dependence of the FWHM extracted from the magnetic Bragg peaks in Figs. 2(a-b) of the main article. The dashed line denotes the average value. Single-crystal neutron diffraction patterns measured at CORELLI at 1.06 GPa and **d** at 45 mK, **e** at 2.1 K, and **f** their difference. The observed powder rings at T=45 mK and 2.1 K originate from the cytop glue, the CuBe pressure cell and the sample holder. Error bars represent one standard deviation.

Supplementary Note 5: Single-crystal neutron diffraction measurements under pressure

Supplementary Figs. 5(a-b) show the sample assembly used for neutron diffraction measurements with an overall sample mosaic of 1°. As indicated in Supplementary Fig. 5(c), there is no evidence of the magnetic Bragg peak broadening when approaching the critical point. Moreover, Supplementary Figs. 5(d-e) are the diffraction patterns measured at P=1.06 GPa ($\sim P_c$) and T=45 mK and 2.1 K, respectively. Their temperature difference in Supplementary Fig. 5(f) does not reveal any incommensurate magnetic Bragg peak or diffuse scattering over a wide range of reciprocal space.

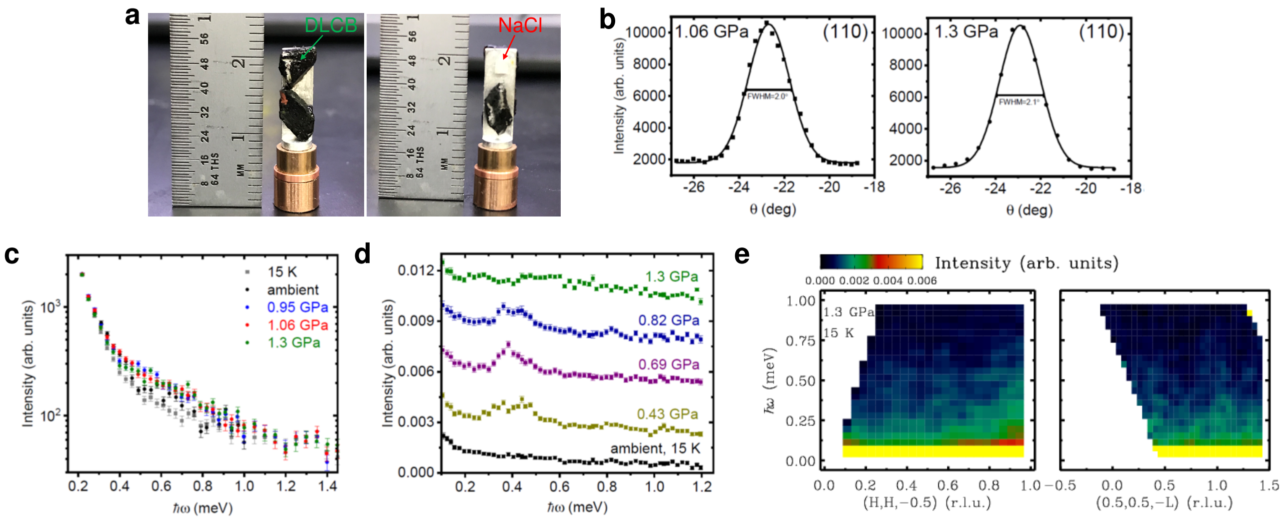

Supplementary Figure 6: **Single-crystal neutron inelastic neutron measurements under pressure.** **a** Photographs of three co-aligned deuterated single crystals of DLCB glued on an aluminum sample holder. A piece of NaCl single crystal was also mounted for calibration of pressure. **b** The rocking-curve scans of the nuclear Bragg reflection (1 1 0) measured at P=1.06 and 1.3 GPa, respectively. Full width at half maximum (FWHM) of the overall sample mosaic is about 2°. Raw INS data as a function of energy transfer measured at **c** MACS and **d** CNCS, respectively. For clarity, the CNCS data are shifted upwards. **e** False-color maps of the excitation spectra as a function of energy and wavevector transfer along two high-symmetry directions (H,H,-0.5) and (0.5,0.5,-L) in the reciprocal space, respectively. High-resolution inelastic neutron scattering data were collected at CNCS and T=15 K and P=1.3 GPa. Error bars represent one standard deviation.

Supplementary Note 6: Single-crystal inelastic neutron measurements under pressure

Supplementary Figs. 6(a-b) show the sample assembly used for the inelastic neutron scattering (INS) with an overall sample mosaic of 2°. Supplementary Figs. 6(c-d) show the raw INS data as a function of energy transfer collected at MACS and CNCS, respectively. Supplementary Fig. 6(e) shows the scattering intensity as a function of energy and wavevector transfer along two high-symmetry directions at P=1.3 GPa and T=15 K.

Atom	x	sx	y	sy	z	sz	B	sB	occ.	socc.
Cu	0.77543	0.00071	0.92327	0.00071	0.21580	0.00039	1.54585	0.10822	1.0	0.0
Br1	0.74204	0.00080	0.81510	0.00082	0.03257	0.00044	1.35615	0.11432	1.0	0.0
Br2	0.83970	0.00081	1.21133	0.00078	0.22144	0.00043	1.24689	0.11588	1.0	0.0
Br3	0.57918	0.00081	0.98447	0.00080	0.35779	0.00044	1.42819	0.11951	1.0	0.0
Br4	0.93329	0.00081	0.69406	0.00078	0.24263	0.00043	1.37262	0.11657	1.0	0.0
C1	0.26546	0.00093	0.92405	0.00090	0.13140	0.00049	1.78224	0.13199	1.0	0.0
D1A	0.23829	0.00103	0.86286	0.00102	0.18950	0.00056	2.86770	0.15318	1.0	0.0
D1B	0.20084	0.00097	1.04466	0.00103	0.14527	0.00055	2.69356	0.15561	1.0	0.0
D1C	0.40911	0.00100	0.93747	0.00098	0.12250	0.00054	2.53545	0.14450	1.0	0.0
C2	0.25267	0.00090	0.63283	0.00088	0.00372	0.00053	1.58151	0.12354	1.0	0.0
D2A	0.39895	0.00103	0.63337	0.00107	0.00323	0.00058	3.09242	0.24852	1.0	0.0
D2B	0.19940	0.00098	0.56387	0.00098	-0.06911	0.00055	2.02689	0.23486	0.96162	0.01668
H2B	0.19940	0.00098	0.56387	0.00098	-0.06911	0.00055	2.02689	0.23486	0.03838	0.01668
D2C	0.20493	0.00106	0.58039	0.00109	0.06319	0.00061	3.39934	0.26269	1.0	0.0
C4	0.20823	0.00082	0.46358	0.00080	0.53857	0.00047	1.33011	0.11759	1.0	0.0
D4	0.16705	0.00096	0.46800	0.00097	0.61399	0.00057	2.38775	0.23039	0.99338	0.01670
H4	0.16705	0.00096	0.46800	0.00097	0.61399	0.00057	2.38775	0.23039	0.00662	0.01670
C5	0.19591	0.00081	0.31936	0.00082	0.45260	0.00046	1.42221	0.11653	1.0	0.0
C6	0.26204	0.00083	0.33690	0.00083	0.36147	0.00047	1.56844	0.12380	1.0	0.0
D6	0.25344	0.00102	0.22767	0.00101	0.29346	0.00055	3.04541	0.20798	1.0	0.0
C7	0.33241	0.00080	0.48930	0.00083	0.35159	0.00044	1.49274	0.12198	1.0	0.0
C8	0.34088	0.00085	0.62549	0.00085	0.44000	0.00046	1.58656	0.12600	1.0	0.0
D8	0.39318	0.00097	0.75234	0.00096	0.44173	0.00053	2.63030	0.19173	1.0	0.0
C9	0.11642	0.00094	0.15675	0.00088	0.45973	0.00053	1.61854	0.11982	1.0	0.0
D9A	0.07118	0.00103	0.16595	0.00104	0.53671	0.00061	3.18505	0.25341	1.0	0.0
D9B	0.21288	0.00118	0.05759	0.00119	0.43991	0.00064	3.51021	0.27298	0.98288	0.01771
H9B	0.21288	0.00118	0.05759	0.00119	0.43991	0.00064	3.51021	0.27298	0.01712	0.01771
D9C	0.00466	0.00113	0.11599	0.00107	0.40732	0.00060	2.84526	0.26361	0.96804	0.01808
H9C	0.00466	0.00113	0.11599	0.00107	0.40732	0.00060	2.84526	0.26361	0.03196	0.01808
C10	0.40100	0.00092	0.50452	0.00096	0.25320	0.00049	1.63151	0.12285	1.0	0.0
D10A	0.43310	0.00113	0.63364	0.00119	0.25577	0.00062	3.92421	0.27533	1.0	0.0
D10B	0.30499	0.00110	0.45045	0.00114	0.19123	0.00064	3.55626	0.26495	1.0	0.0
D10C	0.52272	0.00120	0.43513	0.00119	0.23108	0.00065	3.65799	0.28548	0.97290	0.01810
H10C	0.52272	0.00120	0.43513	0.00119	0.23108	0.00065	3.65799	0.28548	0.02710	0.01810
N1	0.19200	0.00066	0.81119	0.00063	0.03071	0.00035	1.58816	0.09308	1.0	0.0
D1D	0.05307	0.00114	0.80947	0.00112	0.03284	0.00061	2.05951	0.26592	0.89194	0.01796
H1D	0.05307	0.00114	0.80947	0.00112	0.03284	0.00061	2.05951	0.26592	0.10806	0.01796
D1E	0.22201	0.00119	0.86417	0.00119	-0.02677	0.00065	3.19634	0.28282	0.93994	0.01798
H1E	0.22201	0.00119	0.86417	0.00119	-0.02677	0.00065	3.19634	0.28282	0.06006	0.01798
N3	0.28046	0.00059	0.60961	0.00063	0.52805	0.00035	1.56476	0.08978	1.0	0.0
D3	0.29194	0.00116	0.71217	0.00118	0.59101	0.00067	2.38325	0.29533	0.88304	0.01753
H3	0.29194	0.00116	0.71217	0.00118	0.59101	0.00067	2.38325	0.29533	0.11696	0.01753

Supplementary Table 2: Refined atomic coordinates, the isotropic displacement and occupancy factors of deuterated $C_9H_{18}N_2CuBr_4$ from single-crystal neutron diffraction data collected at T=5 K and ambient pressure.

Atom	x	sx	y	sy	z	sz	B	sB	occ.	socc.
Cu	0.76519	0.00762	0.89628	0.00840	0.21638	0.00354	1.54585	0.00000	1.0	0.0
Br1	0.79761	0.01055	0.82513	0.00796	0.03670	0.00333	1.35615	0.00000	1.0	0.0
Br2	0.82603	0.00741	1.19333	0.00767	0.25380	0.00364	1.24689	0.00000	1.0	0.0
Br3	0.59041	0.00834	0.98901	0.00788	0.36294	0.00383	1.42819	0.00000	1.0	0.0
Br4	0.94866	0.00735	0.61554	0.00817	0.24801	0.00331	1.37262	0.00000	1.0	0.0
C1	0.21132	0.01072	1.02196	0.00972	0.15332	0.00331	1.78224	0.00000	1.0	0.0
D1A	0.21132	0.01072	0.93457	0.01170	0.18335	0.00413	2.86770	0.00000	1.0	0.0
D1B	0.32745	0.01301	1.07790	0.00913	0.15834	0.00343	2.69356	0.00000	1.0	0.0
D1C	0.43504	0.00743	0.90916	0.01014	0.13226	0.00338	2.53545	0.00000	1.0	0.0
C2	0.30753	0.00981	0.65071	0.00864	0.03007	0.00338	1.58151	0.00000	1.0	0.0
D2A	0.43143	0.00999	0.64651	0.00894	0.02247	0.00403	3.09242	0.00000	1.0	0.0
D2B	0.16208	0.00825	0.63700	0.00862	-0.06121	0.00377	2.02689	0.00000	0.96162	0.00000
H2B	0.16208	0.00825	0.63700	0.00862	-0.06121	0.00377	2.02689	0.00000	0.03838	0.00000
D2C	0.18801	0.00900	0.53183	0.00869	0.04907	0.00416	3.39934	0.00000	1.0	0.0
C4	0.26402	0.00944	0.44515	0.00888	0.52481	0.00299	1.33011	0.00000	1.0	0.0
D4	0.13948	0.00873	0.53665	0.00935	0.60553	0.00327	2.38775	0.00000	0.99338	0.00000
H4	0.13948	0.00873	0.53665	0.00935	0.60553	0.00327	2.38775	0.00000	0.00662	0.00000
C5	0.22346	0.00840	0.29715	0.00890	0.44284	0.00413	1.42221	0.00000	1.0	0.0
C6	0.25225	0.00685	0.30100	0.00744	0.37198	0.00436	1.56844	0.00000	1.0	0.0
D6	0.28488	0.00964	0.22476	0.00796	0.27763	0.00425	3.04541	0.00000	1.0	0.0
C7	0.33563	0.00973	0.47845	0.00858	0.36602	0.00383	1.49274	0.00000	1.0	0.0
C8	0.35995	0.00995	0.74013	0.00924	0.46241	0.00379	1.58656	0.00000	1.0	0.0
D8	0.40057	0.00896	0.67265	0.00789	0.42011	0.00351	2.63030	0.00000	1.0	0.0
C9	0.11950	0.00942	0.10303	0.00872	0.45582	0.00499	1.61854	0.00000	1.0	0.0
D9A	0.08494	0.00870	0.14737	0.00807	0.54062	0.00563	3.18505	0.00000	1.0	0.0
D9B	0.23623	0.01180	0.05677	0.01040	0.39199	0.00438	3.51021	0.00000	0.98288	0.00000
H9B	0.23623	0.01180	0.05677	0.01040	0.39199	0.00438	3.51021	0.00000	0.01712	0.00000
D9C	-0.00208	0.01126	0.14443	0.00894	0.41115	0.00410	2.84526	0.00000	0.96804	0.00000
H9C	-0.00208	0.01126	0.14443	0.00894	0.41115	0.00410	2.84526	0.00000	0.03196	0.00000
C10	0.44327	0.00890	0.46540	0.00778	0.22832	0.00370	1.63151	0.00000	1.0	0.0
D10A	0.39990	0.00897	0.70659	0.00858	0.27736	0.00511	3.92421	0.00000	1.0	0.0
D10B	0.26790	0.00913	0.48022	0.00933	0.19936	0.00402	3.55626	0.00000	1.0	0.0
D10C	0.55420	0.01332	0.49802	0.00911	0.21636	0.00472	3.65799	0.00000	0.97290	0.00000
H10C	0.55420	0.01332	0.49802	0.00911	0.21636	0.00472	3.65799	0.00000	0.02710	0.00000
N1	0.18420	0.00610	0.83015	0.00570	0.04336	0.00237	1.58816	0.00000	1.0	0.0
D1D	0.02982	0.01055	0.93222	0.01000	0.02961	0.00387	2.05951	0.00000	0.89194	0.00000
H1D	0.02982	0.01055	0.93222	0.01000	0.02961	0.00387	2.05951	0.00000	0.10806	0.00000
D1E	0.21768	0.01040	0.89751	0.00968	-0.05359	0.00447	3.19634	0.00000	0.93994	0.00000
H1E	0.21768	0.01040	0.89751	0.00968	-0.05359	0.00447	3.19634	0.00000	0.06006	0.00000
N3	0.31012	0.00586	0.62369	0.00698	0.51679	0.00278	1.56476	0.00000	1.0	0.0
D3	0.33343	0.00923	0.71055	0.00945	0.58486	0.00486	2.38325	0.00000	0.88304	0.00000
H3	0.33343	0.00923	0.71055	0.00945	0.58486	0.00486	2.38325	0.00000	0.11696	0.00000

Supplementary Table 3: Refined atomic coordinates, the isotropic displacement and occupancy factors of deuterated $C_9H_{18}N_2CuBr_4$ from single-crystal neutron diffraction data collected at T=5 K and 0.88 GPa.

Atom	x	sx	y	sy	z	sz	B	sB	occ.	socc.
Cu	0.72116	0.00652	0.91553	0.00738	0.21533	0.00326	1.54585	0.00000	1.0	0.0
Br1	0.77741	0.00672	0.85210	0.00731	0.04806	0.00161	1.35615	0.00000	1.0	0.0
Br2	0.85305	0.00760	1.18237	0.01100	0.24630	0.00220	1.24689	0.00000	1.0	0.0
Br3	0.54717	0.00976	0.98135	0.01026	0.37493	0.00215	1.42819	0.00000	1.0	0.0
Br4	0.95082	0.00613	0.58115	0.00885	0.24664	0.00245	1.37262	0.00000	1.0	0.0
C1	0.35921	0.00538	0.87674	0.00596	0.10226	0.00197	1.78224	0.00000	1.0	0.0
D1A	0.28790	0.00647	0.79651	0.00528	0.16883	0.00222	2.86770	0.00000	1.0	0.0
D1B	0.09575	0.00652	1.02895	0.00652	0.12403	0.00214	2.69356	0.00000	1.0	0.0
D1C	0.45650	0.00535	1.03709	0.00599	0.13354	0.00163	2.53545	0.00000	1.0	0.0
C2	0.26865	0.00741	0.62032	0.00636	-0.01745	0.00272	1.58151	0.00000	1.0	0.0
D2A	0.38350	0.00628	0.66743	0.00482	0.05514	0.00270	3.09242	0.00000	1.0	0.0
D2B	0.16371	0.00560	0.61298	0.00533	-0.10875	0.00232	2.02689	0.00000	0.96162	0.00000
H2B	0.16371	0.00560	0.61298	0.00533	-0.10875	0.00232	2.02689	0.00000	0.03838	0.00000
D2C	0.20121	0.00429	0.43494	0.00546	0.02908	0.00155	3.39934	0.00000	1.0	0.0
C4	0.23700	0.00559	0.50583	0.00428	0.58906	0.00215	1.33011	0.00000	1.0	0.0
D4	0.17043	0.00589	0.46289	0.00508	0.67163	0.00220	2.38775	0.00000	0.99338	0.00000
H4	0.17043	0.00589	0.46289	0.00508	0.67163	0.00220	2.38775	0.00000	0.00662	0.00000
C5	0.15198	0.00618	0.22871	0.00600	0.43161	0.00158	1.42221	0.00000	1.0	0.0
C6	0.42955	0.00548	0.29626	0.00669	0.33033	0.00182	1.56844	0.00000	1.0	0.0
D6	0.41133	0.00646	0.26108	0.00788	0.37537	0.00186	3.04541	0.00000	1.0	0.0
C7	0.25610	0.00584	0.39322	0.00813	0.34667	0.00245	1.49274	0.00000	1.0	0.0
C8	0.34992	0.00525	0.61409	0.00531	0.42126	0.00185	1.58656	0.00000	1.0	0.0
D8	0.41135	0.00580	0.82827	0.00666	0.40489	0.00200	2.63030	0.00000	1.0	0.0
C9	0.03685	0.00697	0.13002	0.00592	0.43439	0.00206	1.61854	0.00000	1.0	0.0
D9A	0.03778	0.00632	0.20659	0.00612	0.52229	0.00202	3.18505	0.00000	1.0	0.0
D9B	0.48067	0.00818	0.00563	0.00845	0.47857	0.00287	3.51021	0.00000	0.98288	0.00000
H9B	0.48067	0.00818	0.00563	0.00845	0.47857	0.00287	3.51021	0.00000	0.01712	0.00000
D9C	-0.09514	0.00660	0.15676	0.00748	0.34913	0.00169	2.84526	0.00000	0.96804	0.00000
H9C	-0.09514	0.00660	0.15676	0.00748	0.34913	0.00169	2.84526	0.00000	0.03196	0.00000
C10	0.39377	0.00648	0.62233	0.00563	0.25668	0.00200	1.63151	0.00000	1.0	0.0
D10A	0.46718	0.00549	0.63500	0.00675	0.20118	0.00226	3.92421	0.00000	1.0	0.0
D10B	0.29913	0.00794	0.32951	0.00751	0.24481	0.00247	3.55626	0.00000	1.0	0.0
D10C	0.73913	0.00832	0.45026	0.00698	0.23069	0.00273	3.65799	0.00000	0.97290	0.00000
H10C	0.73913	0.00832	0.45026	0.00698	0.23069	0.00273	3.65799	0.00000	0.02710	0.00000
N1	0.19284	0.00423	0.82515	0.00476	0.01727	0.00126	1.58816	0.00000	1.0	0.0
D1D	0.00322	0.00747	0.90986	0.00754	0.04875	0.00238	2.05951	0.00000	0.89194	0.00000
H1D	0.00322	0.00747	0.90986	0.00754	0.04875	0.00238	2.05951	0.00000	0.10806	0.00000
D1E	0.19064	0.00947	0.96748	0.00574	-0.03502	0.00207	3.19634	0.00000	0.93994	0.00000
H1E	0.19064	0.00947	0.96748	0.00574	-0.03502	0.00207	3.19634	0.00000	0.06006	0.00000
N3	0.26636	0.00495	0.56009	0.00369	0.50126	0.00178	1.56476	0.00000	1.0	0.0
D3	0.35141	0.00721	0.69156	0.00783	0.57395	0.00236	2.38325	0.00000	0.88304	0.00000
H3	0.35141	0.00721	0.69156	0.00783	0.57395	0.00236	2.38325	0.00000	0.11696	0.00000

Supplementary Table 4: Refined atomic coordinates, the isotropic displacement and occupancy factors of deuterated $C_9H_{18}N_2CuBr_4$ from single-crystal neutron diffraction data collected at T=5 K and 1.06 GPa.

Atom	x	sx	y	sy	z	sz	B	sB	occ.	socc.
Cu	0.73642	0.00779	0.90416	0.00638	0.21851	0.00312	1.54585	0.00000	1.0	0.0
Br1	0.78401	0.00867	0.83679	0.00746	0.04342	0.00314	1.35615	0.00000	1.0	0.0
Br2	0.85936	0.00794	1.17366	0.00705	0.24533	0.00295	1.24689	0.00000	1.0	0.0
Br3	0.54364	0.00865	0.98439	0.00746	0.37280	0.00313	1.42819	0.00000	1.0	0.0
Br4	0.94067	0.00901	0.57871	0.00750	0.24555	0.003333	1.37262	0.00000	1.0	0.0
C1	0.48324	0.00823	0.83173	0.00737	0.12373	0.00290	1.78224	0.00000	1.0	0.0
D1A	0.30183	0.01034	0.84200	0.00695	0.22534	0.00391	2.86770	0.00000	1.0	0.0
D1B	0.13809	0.01043	1.08278	0.00924	0.15081	0.00368	2.69356	0.00000	1.0	0.0
D1C	0.61178	0.01249	0.91369	0.00752	0.11943	0.00427	2.53545	0.00000	1.0	0.0
C2	0.27452	0.00793	0.62892	0.00771	-0.00992	0.00356	1.58151	0.00000	1.0	0.0
D2A	0.34053	0.00969	0.62407	0.00829	0.03886	0.00352	3.09242	0.00000	1.0	0.0
D2B	0.17765	0.01048	0.71780	0.00757	-0.15043	0.00356	2.02689	0.00000	0.96162	0.00000
H2B	0.17765	0.01048	0.71780	0.00757	-0.15043	0.00356	2.02689	0.00000	0.03838	0.00000
D2C	-0.19767	0.01082	0.62223	0.00929	0.01494	0.00374	3.39934	0.00000	1.0	0.0
C4	0.24515	0.00878	0.56301	0.00931	0.57828	0.00405	1.33011	0.00000	1.0	0.0
D4	-0.00615	0.01010	0.31246	0.00920	0.63951	0.00329	2.38775	0.00000	0.99338	0.00000
H4	-0.00615	0.01010	0.31246	0.00920	0.63951	0.00329	2.38775	0.00000	0.00662	0.00000
C5	0.21106	0.00938	0.20658	0.00798	0.44588	0.00346	1.42221	0.00000	1.0	0.0
C6	0.35432	0.01112	0.34924	0.00880	0.35366	0.00396	1.56844	0.00000	1.0	0.0
D6	0.49239	0.00913	0.29784	0.00708	0.43429	0.00355	3.04541	0.00000	1.0	0.0
C7	0.22883	0.01000	0.44056	0.00877	0.33213	0.00428	1.49274	0.00000	1.0	0.0
C8	0.39589	0.00946	0.63094	0.00874	0.41229	0.00316	1.58656	0.00000	1.0	0.0
D8	0.39016	0.00955	0.78930	0.01190	0.46632	0.00301	2.63030	0.00000	1.0	0.0
C9	0.05706	0.01170	0.14455	0.01165	0.42296	0.00337	1.61854	0.00000	1.0	0.0
D9A	-0.02341	0.01059	0.31620	0.00867	0.56316	0.00360	3.18505	0.00000	1.0	0.0
D9B	0.30190	0.01188	-0.00574	0.01281	0.49369	0.00379	3.51021	0.00000	0.98288	0.00000
H9B	0.30190	0.01188	-0.00574	0.01281	0.49369	0.00379	3.51021	0.00000	0.01712	0.00000
D9C	0.07011	0.01035	0.16731	0.00852	0.38437	0.00395	2.84526	0.00000	0.96804	0.00000
H9C	0.07011	0.01035	0.16731	0.00852	0.38437	0.00395	2.84526	0.00000	0.03196	0.00000
C10	0.33405	0.01224	0.61144	0.01399	0.25214	0.00461	1.63151	0.00000	1.0	0.0
D10A	0.52001	0.01130	0.49582	0.00878	0.20851	0.00345	3.92421	0.00000	1.0	0.0
D10B	0.32617	0.01289	0.34199	0.00899	0.25392	0.00428	3.55626	0.00000	1.0	0.0
D10C	0.74512	0.01515	0.54819	0.00852	0.27263	0.00450	3.65799	0.00000	0.97290	0.00000
H10C	0.74512	0.01515	0.54819	0.00852	0.27263	0.00450	3.65799	0.00000	0.02710	0.00000
N1	0.14973	0.00735	0.83919	0.00634	0.02644	0.00207	1.58816	0.00000	1.0	0.0
D1D	0.08842	0.00970	1.00993	0.01148	0.04366	0.00410	2.05951	0.00000	0.89194	0.00000
H1D	0.08842	0.00970	1.00993	0.01148	0.04366	0.00410	2.05951	0.00000	0.10806	0.00000
D1E	0.25622	0.01141	0.97369	0.00934	-0.02976	0.00416	3.19634	0.00000	0.93994	0.00000
H1E	0.25622	0.01141	0.97369	0.00934	-0.02976	0.00416	3.19634	0.00000	0.06006	0.00000
N3	0.24624	0.00650	0.56322	0.00594	0.51621	0.00245	1.56476	0.00000	1.0	0.0
D3	0.38773	0.00967	0.65489	0.01121	0.61983	0.00370	2.38325	0.00000	0.88304	0.00000
H3	0.38773	0.00967	0.65489	0.01121	0.61983	0.00370	2.38325	0.00000	0.11696	0.00000

Supplementary Table 5: Refined atomic coordinates, the isotropic displacement and occupancy factors of deuterated $C_9H_{18}N_2CuBr_4$ from single-crystal neutron diffraction data collected at T=5 K and 1.3 GPa.

	ambient	0.88 GPa	1.06 GPa	1.3 GPa
$d_{\mathrm{Cu}\cdots\mathrm{Cu}}$ (Å)—J_{rung}	8.622(11)	8.345(10)	8.186(10)	8.075(9)
$d_{\mathrm{Cu}\cdots\mathrm{Cu}}$ (Å)—J_{leg}	8.268(12)	8.148(13)	8.083(14)	8.053(14)
$d_{\mathrm{Cu}\cdots\mathrm{Cu}}$ (Å)—J_{int}	7.261(12)	7.402(11)	7.521(12)	7.552(10)
$d_{\mathrm{Cu}\cdots\mathrm{Cu}}$ (Å)—J'_{layer}	7.618(12)	7.534(13)	7.473(11)	7.102(12)
$d_{\mathrm{Cu}\cdots\mathrm{Cu}}$ (Å)—J''_{layer}	7.454(11)	7.206(12)	7.178(13)	7.108(13)
$d_{\mathrm{Br3}\cdots\mathrm{Br3}}$ (Å)—J_{rung}	4.031(12)	3.899(10)	3.366(10)	3.418(9)
$d_{\mathrm{Br2}\cdots\mathrm{Br4}}$ (Å)—J_{leg}	3.963(10)	3.564(11)	3.294(11)	3.305(9)
$d_{\mathrm{Br1}\cdots\mathrm{Br1}}$ (Å)—J_{int}	5.122(13)	4.476(12)	4.412(13)	4.443(13)
$d_{\mathrm{Br1}\cdots\mathrm{Br1}}$ (Å)—J'_{layer}	5.015(14)	5.418(13)	4.892(12)	4.920(13)
$d_{\mathrm{Br3}\cdots\mathrm{Br4}}$ (Å)—J''_{layer}	5.353(12)	5.442(11)	5.297(10)	5.310(12)
$\theta_{\mathrm{Cu-Br3}\cdots\mathrm{Br3}}$ (°)—J_{rung}	156.8(4)	157.7(5)	152.2(5)	158.8(4)
$\theta_{\mathrm{Cu-Br2}\cdots\mathrm{Br4}}$, $\theta_{\mathrm{Cu-Br4}\cdots\mathrm{Br2}}$ (°)—J_{leg}	177.3(3),138.0(3)	166.5(3),136.8(4)	170.4(4),139.2(4)	167.9(3),137.5(3)
$\theta_{\mathrm{Cu-Br1}\cdots\mathrm{Br1}}$ (°)—J_{int}	93.6(3)	109.7(4)	115.8(4)	114.8(3)
$\theta_{\mathrm{Cu-Br1}\cdots\mathrm{Br1}}$ (°)—J'_{layer}	101.3(3)	96.9(3)	104.3(3)	97.0(3)
$\theta_{\mathrm{Cu-Br3}\cdots\mathrm{Br4}}$, $\theta_{\mathrm{Cu-Br4}\cdots\mathrm{Br3}}$ (°)—J''_{layer}	110.3(3),101.0(3)	102.5(3),92.1(4)	102.5(4),87.7(4)	101.1(3),88.2(3)

Supplementary Table 6: The Cu· · ·Cu separation distance, Br· · ·Br contact distances and Cu-Br· · ·Br bridging angles for Cu-Br· · ·Br-Cu superexchange paths of J_{rung}, J_{leg}, J_{int}, J'_{layer} and J''_{layer} at several representative pressures. The bromide ions are enumerated in Fig. 4(b).

Hamiltonian parameter	ambient	1.03 GPa	1.3 GPa
J_{leg} (meV)	0.62	0.91	1.03
J_{rung} (meV)	0.66	0.97	1.1
J_{int} (meV)	0.20	0.13	0.05
$\alpha = J_{\mathrm{int}}/J_{\mathrm{leg}}$	0.32	0.14	0.05
λ	0.87	0.87	0.87

Supplementary Table 7: Alteration of the Hamiltonian parameters and the exchange coupling ratio α between the inter-ladder and intra-ladder couplings at different pressures obtained by quantum Monte Carlo calculations.

References

[1] Nohadani, O., Wessel, S., Normand, B. & Haas, S. Universal scaling at field-induced magnetic phase transitions. *Phys. Rev. B* **69**, 220402(R) (2004).

[2] Sebastian, S. E. *et al.* Characteristic Bose-Einstein condensation scaling close to a quantum critical point in $BaCuSi_2O_6$. *Phys. Rev. B* **72**, 100404(R) (2005).

[3] Winter, S. M. *et al.* Breakdown of magnons in a strongly spin-orbital coupled magnet. *Nat. Commun.* **8**, 1152 (2017).

[4] Turnbull, M. M., Landee, C. P. & Wells,B. M. Magnetic exchange interactions in tetrabromocuprate compounds. *Coord. Chem. Rev.* **249**, 2567 (2005).
